# Association of maternal overweight and gestational diabetes mellitus with offspring adiposity trajectory: from birth to early adolescence

**DOI:** 10.1007/s00125-025-06468-6

**Published:** 2025-06-17

**Authors:** Yuzhi Deng, Claudia H. T. Tam, Aimin Yang, Mai Shi, Lai Yuk Yuen, Noel Y. H. Ng, Atta Y. T. Tsang, Kit Ying Tsoi, Risa Ozaki, Albert M. Li, Elaine Chow, Lai Ling Hui, Juliana C. N. Chan, Chi Chiu Wang, Wing Hung Tam, Ronald C. W. Ma

**Affiliations:** 1 Department of Medicine and Therapeutics, The Chinese University of Hong Kong, Hong Kong, China; 2 Li Ka Shing Institute of Health Sciences, The Chinese University of Hong Kong, Hong Kong, China; 3 Hong Kong Institute of Diabetes and Obesity, The Chinese University of Hong Kong, Hong Kong, China; 4 Department of Obstetrics and Gynaecology, The Chinese University of Hong Kong, Hong Kong, China; 5 Department of Paediatrics, The Chinese University of Hong Kong, Hong Kong, China; 6 Department of Food Science and Nutrition, The Hong Kong Polytechnic University, Hong Kong, China; 7 CUHK Medical Centre, Shatin, Hong Kong, China

**Keywords:** Gestational diabetes mellitus, Growth trajectory, Maternal BMI, Maternal hyperglycaemia, Offspring adiposity

## Abstract

**Aims/hypothesis:**

We aimed to examine offspring adiposity trajectories from birth to age 9–14 years and to assess the joint associations of maternal overweight and gestational diabetes mellitus (GDM) with these trajectories.

**Methods:**

This is a prospective cohort study with 564 mother–child dyads from the Hyperglycemia and Adverse Pregnancy Outcome study Hong Kong field centre. Assessments and anthropometric measurements were taken during pregnancy, at delivery and at median ages of 7 and 10 years postpartum. Offspring adiposity was primarily assessed using sum of skinfold thickness. We used linear mixed-effect models to evaluate the independent and joint associations of maternal overweight and GDM with the offspring adiposity trajectories, and applied group-based trajectory modelling to identify distinct patterns of adiposity development based on both statistical indices and clinical interpretability.

**Results:**

Offspring skinfold thickness trajectories varied significantly based on maternal overweight and GDM (*p*<0.05). Group-based trajectory modelling identified two trajectory groups for skinfold thickness: 52.1% with slow increase and 47.9% with rapid increase. Combined maternal overweight and GDM was associated with 6.90-fold increased risk (95% CI 1.89, 33.32; *p*=0.006) of the rapidly increasing trajectory. Linear mixed-effect model analysis showed greater increases in skinfold thickness among offspring of mothers with either condition, with the highest trajectory observed in offspring of mothers with both conditions (β 1.62; 95% CI 0.69, 2.54; *p*=0.001).

**Conclusions/interpretation:**

Maternal overweight and GDM are independently and jointly associated with rapidly increasing adiposity trajectories from birth to early adolescence. The findings underscore the importance of considering both maternal metabolic conditions when evaluating offspring adiposity risk.

**Graphical Abstract:**

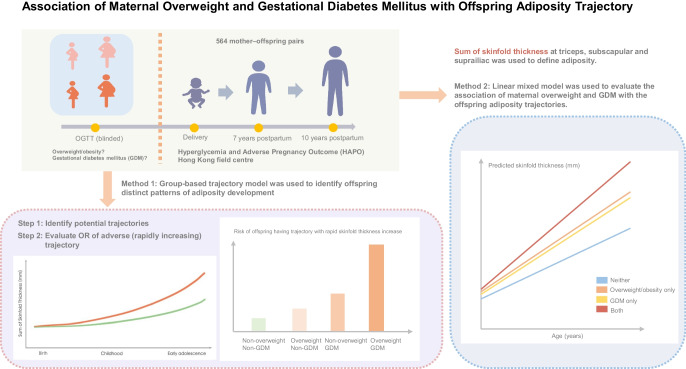

**Supplementary Information:**

The online version contains peer-reviewed but unedited supplementary material available at 10.1007/s00125-025-06468-6.


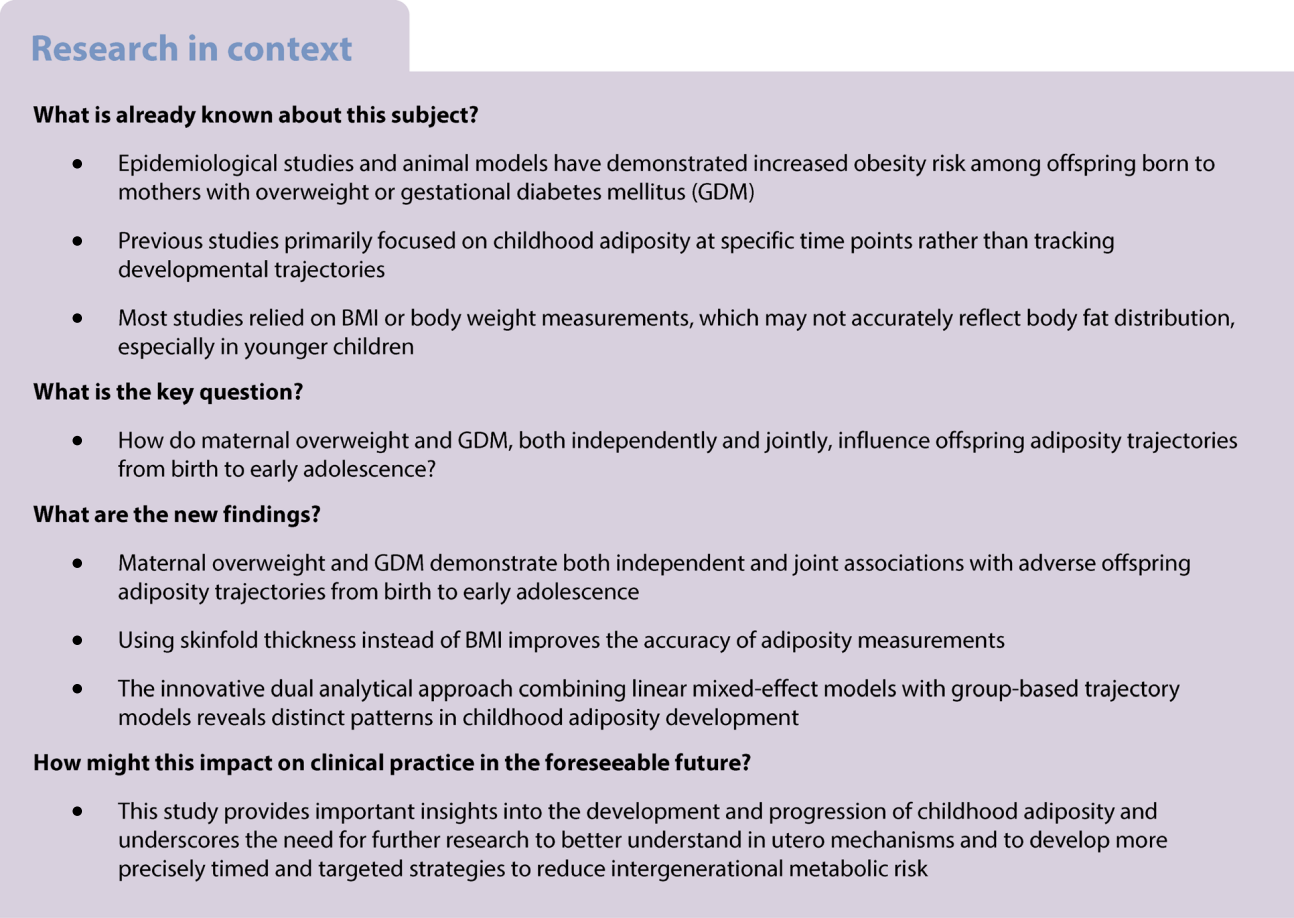


## Introduction

Childhood overweight or obesity is a significant global health concern, with its prevalence having increased dramatically across all world regions in recent decades [1]. In particular, east Asia has experienced one of the largest absolute increases in the number of children and adolescents with obesity globally [2]. In Hong Kong, recent surveillance data show that overweight or obesity affects approximately 19.0% of primary school students and 21.3% of secondary school students [3]. These rates are particularly concerning given that Asian populations tend to develop adverse metabolic outcomes at lower BMI thresholds compared with Western populations [4]. From a life course perspective, the negative health impacts of overweight and obesity accumulate over time, leading to worse cardiometabolic profiles in adulthood. Children with obesity are more likely to remain obese into adulthood, increasing their risks of metabolic syndrome, type 2 diabetes and coronary heart disease [1]. Identifying early-life indicators and understanding postnatal growth patterns involved in later obesity are crucial for developing effective preventive strategies.

Maternal prenatal nutritional characteristics, such as preconceptional obesity, excessive gestational weight gain and gestational diabetes mellitus (GDM), have been identified as significant contributors to childhood obesity [5, 6]. This relationship has been widely supported by both epidemiological and animal studies, which have consistently documented the heightened risk of obesity among offspring born to mothers with overweight or GDM [7, 8]. While maternal obesity and GDM may both contribute to offspring obesity, they operate through distinct biological pathways. Maternal obesity primarily influences fetal development through altered adipokine profiles and inflammatory mediators [8], whereas GDM affects offspring metabolism primarily through fetal hyperinsulinaemia and altered glucose–insulin homeostasis [9]. These different mechanistic pathways may interact to create potentially synergistic effects on offspring adiposity development. However, previous studies have often focused on the independent associations of maternal glucose levels measured mid-pregnancy, modelled both as GDM vs no GDM and along the continuum of observed glucose values, with offspring adiposity, adjusting for maternal BMI [10, 11]. Most epidemiological studies examining the association between maternal BMI or GDM and later offspring outcomes, as well as those examining childhood BMI and cardiometabolic outcomes, have focused on childhood adiposity at specific points in time [6]. However, tracking trajectory rather than single timepoint measurements provides insights into timing and patterns of adiposity accumulation, which may provide important implications for later health outcomes. Also, many studies were generally limited to measurement of childhood BMI or body weight, which may not accurately reflect body fat distribution, potentially leading to misclassification of adiposity status, especially in young children [12, 13]. More direct measures of adiposity, such as dual-energy x-ray absorptiometry (DXA), bioelectrical impedance analysis (BIA) or skinfold thickness measurements, provide a more accurate assessment of body composition. While DXA requires specialised equipment and significant cost, and BIA measurements can be affected by hydration status, skinfold thickness stands out as a practical and reliable method for assessing body composition in children as it measures subcutaneous fat, which accounts for about 40–60% of total body fat.

To address these gaps, we undertook this study to examine the combined association of maternal overweight and GDM with offspring adiposity trajectory from birth to early adolescence in a prospective cohort of mother–child dyads, using sum of skinfold thickness as the main measurement, with the aim of identifying growth patterns strongly associated with childhood overweight/obesity, and thereby informing clinical care and targeted interventions. We hypothesised that children born to mothers with both overweight and GDM would have more rapid adiposity gain, and follow distinct growth trajectories, compared with those born to mothers with either condition alone or without either condition.

## Methods

### Study design

**Fig. 1:**
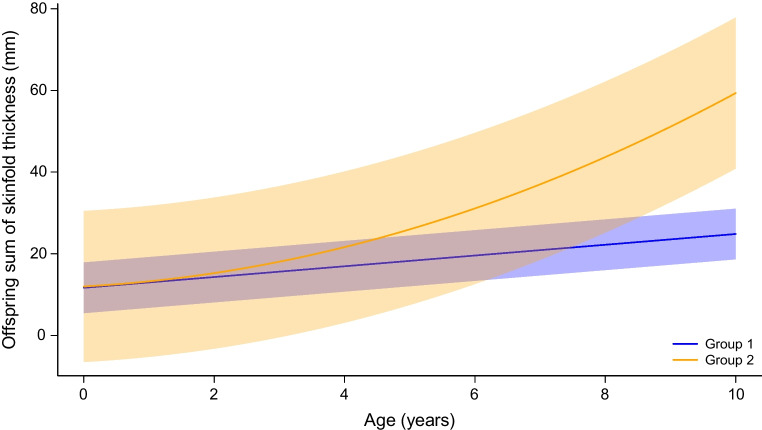
Offspring sum of skinfold trajectory categories from birth to early adolescence from the group-based trajectory model. Two latent trajectory groups with quadratic functions were identified. The blue curve (group 1, *n*=294) is categorised as the slowly increasing skinfold thickness trajectory, and the orange curve (group 2, *n*=270) is categorised as the rapidly increasing skinfold thickness trajectory. Shadow indicates the corresponding 95% CIs

This is a prospective cohort analysis that integrates data from the original Hyperglycemia and Adverse Pregnancy Outcome (HAPO) study conducted between 2000 and 2006 at the Hong Kong field centre and its subsequent follow-up evaluations. The HAPO study, an observational, multinational, blinded, population-based study, investigated associations between maternal plasma glucose level during pregnancy and perinatal outcomes [14]. Initially, 1667 pregnant women with singleton pregnancies were enrolled in the Hong Kong centre and underwent a 75 g OGTT at 24–32 gestational weeks. Both participants and clinicians were blinded to the OGTT results. Exclusion criteria included non-Asian participants and those with missing data on maternal preconceptional weight and glucose level. Maternal ethnicity was determined based on self-reported information collected during the initial enrolment. The first follow-up evaluation was conducted at a median of 6.9 years (interquartile range: 6.7–7.2 years) postpartum (2009–2013) [15]. This intermediate follow-up was uniquely performed at the Hong Kong centre. The second follow-up at a median of 10.1 years (interquartile range: 9.6–11.1 years) postpartum (2013–2016) was part of the multi-centre HAPO Follow-up Study [16]. A total of 564 eligible mother–child pairs attended both follow-up study visits (electronic supplementary material [ESM] Fig. 1).

The study was approved by the New Territories East Cluster–Chinese University of Hong Kong (NTEC-CUHK) Clinical Research Ethics Committee. The study was reported according to the Strengthening the Reporting of Observational Studies in Epidemiology (STROBE) guidelines for cohort studies.

### Data collection

Maternal demographic data, including parity, education and cigarette smoking, and paternal characteristics were collected from standardised questionnaires. Neonatal measurements were obtained by a standardised method by trained research staff within 72 h of delivery, as previously described in the original HAPO study [14].

At follow-up visits, offspring anthropometric measures included weight, height and skinfold thickness. Weight was measured to the nearest 0.1 kg using a calibrated scale, and height was measured twice with a stadiometer to the nearest 0.5 cm. Skinfold thickness at triceps, subscapular and suprailiac was measured by Holtain Tanner/Whitehouse skinfold callipers (Holtain) twice to the nearest 0.1 mm. A third measurement was obtained if results differed by >0.5 kg for weight, >1.0 cm for height or >0.5 mm for skinfold thickness. The mean of the two or three measurements was used for analysis. Demographic data on personal medical history, family history of diabetes, dietary habits and physical activity were collected using structured questionnaires.

### Exposure, outcome and covariates

The primary exposures were maternal preconceptional BMI and GDM. BMI was treated as both a continuous and a categorical variable, with overweight defined as BMI ≥24.0 kg/m^2^ according to Chinese population standards [17]. Preconceptional weight was obtained by participant recall at the first antenatal visit and at recruitment of the original HAPO study. Maternal height was measured at the first antenatal visit. For the maternal glucose analysis, continuous analysis adopted maternal plasma glucose level using the sum of glucose *z* scores at fasting, 1 h and 2 h during the OGTT. GDM was defined post hoc using International Association of Diabetes in Pregnancy Study Groups (IADPSG) criteria using the standard 75 g 2 h OGTT [18], with GDM if any one of the following criteria was met: fasting blood glucose values ≥5.1 mmol/l, 1 h glucose values ≥10.0 mmol/l or 2 h glucose values ≥8.5 mmol/l.

The outcomes included: adiposity measured by sum of skinfold thickness (primary outcome), BMI, sum of the skinfold measurements at each timepoint and sum of skinfold growth (changes in mm per year) from birth to early adolescence.

Maternal and child characteristics that could potentially affect growth patterns were considered as confounders, including maternal age, maternal education level (categorised as higher for over 11 years of education, or lower for 10 years or less), parity (primiparity/multiparity), maternal smoking (yes/no), paternal BMI (at original OGTT), offspring breastfeeding status (yes/no), paternal diabetes status (yes/no) and offspring sex (male/female). Offspring sex was determined based on medical records at birth during the initial HAPO study. A directed acyclic graph (DAG) was built to show hypothesised causal correlations between variables (ESM Fig. 2).

### Statistical analysis

Data were summarised using frequencies and counts for categorical variables and means and SDs for continuous variables. Maternal overweight and GDM were modelled as independent main effects with childhood sum of skinfold thickness trajectory. Joint analyses of maternal overweight and GDM were calculated by creating four categories based on observed HAPO data: (1) non-overweight and non-GDM; (2) overweight and non-GDM; (3) non-overweight and GDM; and (4) overweight and GDM.

To examine both population-level trends and subgroup-specific patterns in adiposity development, we employed two complementary analytical approaches. First, group-based trajectory modelling (GBTM), which assumes heterogeneous groups following different trajectories within a study population, was conducted to identify distinct groups for trajectories of the sum of skinfold thickness [19]. This data-driven approach uses maximum likelihood estimation to identify groups of individuals following similar developmental paths. Censored normal models were specified with age (in years) as the independent variable and repeated measurements of skinfold thickness as the outcome variable. The ideal number of classes for each cohort was established using Akaike’s information criterion (AIC), Bayesian information criterion (BIC) statistics, odds of correct classification (OCC), average posterior probability (APP), the proportion of participants with posterior probability >0.7, the proportion of individuals in each group and clinical interpretation (ESM Table 1). While statistical fit indices suggested better fit for three- or four-class models, we finally selected the two-class model based on clinical interpretability and to ensure sufficient sample size within each maternal exposure category. When the three- and four-class models were examined across different maternal exposure categories, some resulting subgroups contained extremely small numbers of or zero participants, which would limit our ability to conduct robust statistical analysis and draw reliable interpretation. ORs and 95% CIs are presented for the likelihood of being in the specific skinfold thickness trajectory group given a certain exposure variable. Multivariable logistic regression, adjusted for the aforementioned child and maternal covariates, was then conducted to assess the association between maternal GDM and overweight variables and the skinfold thickness trajectory groups. To assess potential additive interaction between maternal overweight and GDM, we calculated the relative excess risk due to interaction (RERI), attributable proportion due to interaction (AP) and synergy index (S) with their 95% CIs.

A linear mixed-effect model (LMM) was used to describe the trajectories for the sum of skinfold thickness. The basic model included random intercept and random slope components, with offspring age (in years) entered as a fixed effect. Maternal overweight and GDM group factors were then included as fixed factors and as interaction terms with offspring age to estimate the increase in skinfold thickness from growth for each category. To account for potential confounding, the model was adjusted for potential covariates including maternal age, education level, parity, smoking, paternal BMI, paternal diabetes status, offspring sex and offspring breastfeeding status.

Additionally, a multivariable linear model and a logistic regression model explored the associations between maternal exposures (specifically, maternal overweight/BMI and GDM status) and childhood adiposity at specific time points. Restricted cubic spline (RCS) models analysed the dose–response relationships between maternal BMI and offspring adiposity risk (defined as skinfold thickness exceeding 85th percentile stratified by age and sex in our population) in GDM and non-GDM subgroups. We used four knots placed at the 5th, 35th, 65th and 95th percentiles of the exposure distribution. For analysis of offspring adiposity at the first follow-up, 961 mother–child dyads were included, with a characteristic comparison of inclusion and exclusion shown in ESM Table 2. Also, the comparison of baseline characteristics between follow-up and lost to follow-up at the second follow-up is shown in ESM Table 3. It should be noted that missing data of covariates were not imputed in our analyses due to the very small proportion (less than 5%), which was not likely to significantly impact our results. Sensitivity analysis was conducted by using BMI trajectory as the adiposity outcome to test the robustness of the original results.

All data analyses were performed using R software (v.4.3.2; https://www.r-project.org/). We used the following R packages: ‘gbmt’ for GBTM, ‘lme4’ for LMM, ‘rms’ for RCS models. Two-sided *p* values of less than 0.05 were considered statistically significant.

## Results

Table 1 displays the characteristics of mothers at the original HAPO study and their offspring at the first and second follow-ups. Among the mothers, 484 (85.8%) were classified as non-overweight and 80 (14.2%) as overweight or obese. The prevalence of GDM in the current analysis was 12.8% (*n*=72).

**Table 1:** Participant characteristics included in final trajectory analysis

Characteristic	Total (*n*=564)	Maternal non-overweight and non-GDM (*n*=425)	Maternal overweight and non-GDM (*n*=67)	Maternal non-overweight and GDM (*n*=59)	Maternal overweight and GDM (*n*=13)	*p*
Maternal characteristics during HAPO study						
Age (years)	31.5±4.6	31.6±4.6	31.8±4.5	32.6±4.4	34.1±4.5	0.025
Preconceptional BMI (kg/m^2^)	21.1±2.9	20.1±1.9	26.3±2.2	20.6±1.7	26.6±2.4	<0.001
Higher education level^a^	527 (96.2)	398 (97.1)	65 (97.0)	52 (89.7)	12 (92.3)	0.041
Parity, primiparity	327 (58.0)	259 (60.9)	34 (50.7)	32 (54.2)	2 (15.4)	0.005
Family history of diabetes	206 (36.5)	156 (36.7)	25 (37.3)	19 (32.2)	6 (46.2)	0.797
Smoker	6 (1.1)	3 (0.7)	0 (0)	2 (3.4)	1 (7.7)	0.039^b^
Paternal characteristics during HAPO study						
Paternal BMI at OGTT (kg/m^2^)	23.3±3.3	23.2±3.2	23.7±3.5	23.5±3.6	22.7±4.2	0.578
Paternal diabetes	18 (3.2)	11 (2.6)	1 (1.5)	4 (6.8)	2 (15.4)	0.028
Offspring characteristics at birth during HAPO study						
Sex, male	297 (52.7)	228 (53.6)	37 (55.2)	28 (47.5)	4 (30.8)	0.323
Weight (kg)	3.2±0.4	3.2±0.4	3.3±0.5	3.3±0.4	3.3±0.4	0.004
Length (cm)	49.2±1.8	49.2±1.8	49.6±1.8	48.9±1.7	49.6±1.7	0.175
Ponderal index at birth (g/cm^3^)	2.7±0.2	2.7±0.2	2.7±0.2	2.8±0.3	2.7±0.2	<0.001
BMI (kg/m^2^)	13.2±1.2	13.1±1.1	13.4±1.2	13.7±1.4	13.6±1.1	<0.001
Gestational week (weeks)	39.5±1.1	39.5±1.1	39.6±1.1	39.4±1.2	39.6±1.1	0.717
Breastfeeding^a^	280 (49.8)	208 (49.2)	30 (44.8)	35 (59.3)	7 (53.9)	0.397
Offspring characteristics during HAPO 7 year follow-up study						
Age (years)	6.9±0.4	7.0±0.4	7.0±0.4	6.8±0.5	7.0±0.4	0.222
Weight (kg)	23.3±4.4	22.8±4.1	25.3±5.8	23.7±3.7	25.5±5.2	<0.001
Height (cm)	124.1±4.9	123.8±4.8	126.2±5.1	124.0±4.6	125.5±4.2	0.002
BMI (kg/m^2^)	15.0±2.2	14.8±2.0	15.8±2.9	15.4±1.9	16.1±2.7	<0.001
Waist circumference (cm)	53.9±5.8	53.3±5.5	56.2±7.2	54.8±5.3	56.9±5.7	<0.001
Hip circumference (cm)	64.5±5.8	63.9±5.5	66.8±7.2	65.0±4.9	67.9±5.8	<0.001
Sum of skinfold (mm)	28.7 ± 13.4	27.3 ± 12.3	33.4 ± 16.9	31.5 ± 14.3	37.4 ± 15.5	<0.001
Body fat percentage (%)	18.9±6.5	18.2±6.1	21.0±8.0	20.1±6.3	23.3±8.1	<0.001
Exercise frequency^a^						0.760
Never	44 (7.8)	31 (7.3)	6 (9.0)	6 (10.2)	1 (7.7)	
Regular	317 (56.3)	246 (58.0)	37 (55.2)	27 (45.7)	7 (53.9)	
Frequent	202 (35.9)	147 (34.7)	24 (35.8)	26 (44.1)	5 (38.5)	
Offspring characteristics during HAPO 10 year follow-up study						
Age (years)	10.4±1.0	10.4±1.0	10.4±0.9	10.4±1.1	10.2±0.9	0.936
Weight (kg)	35.4±8.9	34.6±8.7	38.6±9.9	37.2±8.2	39.0±7.2	0.001
Height (cm)	141.2±8.9	140.6±9.0	143.5±8.3	142.74±8.4	142.0±9.8	0.046
BMI (kg/m^2^)	17.6±3.1	17.3±3.0	18.6±3.6	18.1±2.9	19.4±3.1	0.001
Waist circumference (cm)	61.6±9.1	60.6±8.8	64.8±10.2	63.5±8.3	66.6±7.6	<0.001
Sum of skinfold (mm)	41.3±21.9	39.0±21.1	47.9±25.5	46.4±20.6	58.6±19.9	<0.001
Body fat percentage (%)	19.9±9.1	19.1±8.9	23.6±10.1	20.8±8.3	23.4±7.9	0.001

Data are shown as mean±SD or *n* (%)

^a^Percentages were calculated based on valid responses for each variable (excluding missing data)

^b^With Fisher exact test

We identified two adiposity trajectories in sum of skinfold thickness from birth to age approximately 10 years: 294 children (52.1%, 48.3% girls and 51.7% boys) were classified into the slowly increasing skinfold thickness trajectory, and 270 (47.9%, 46.3% girls and 53.7% boys) into the rapidly increasing skinfold thickness trajectory (Fig. 1). The rapidly increasing skinfold thickness trajectory was chosen as the adverse outcome, serving as a proxy for the risk of childhood adiposity, to reveal clinically interpretable findings. For subsequent analysis, the slowly increasing skinfold thickness trajectory group was used as the reference group.

The associations of maternal overweight and GDM with the odds of being in the rapidly increasing trajectory group are presented in Table 2. After adjustment, maternal overweight (OR 2.04; 95% CI 1.21, 3.48) and GDM (OR 2.95; 95% CI 1.67, 5.34) were positively and independently associated with childhood adiposity outcomes. Continuous associations of maternal BMI or sum of all glucose *z* scores at OGTT with offspring rapidly increasing skinfold thickness trajectory are also seen in ESM Table 4. Table 3 shows the joint associations of maternal overweight and GDM with childhood skinfold thickness trajectories from birth to early adolescence. Children born to mothers categorised as overweight and GDM had the highest odds of being classified into the rapidly increasing skinfold thickness trajectory group, compared with those born to normal-weight and non-GDM mothers (OR 6.90; 95% CI 1.89, 33.32). In the additive interaction analysis between maternal overweight and GDM, we observed RERI=3.04 (95% CI −6.57, 12.64), AP=0.44 (95% CI −0.37, 1.25) and S=2.06 (95% CI 0.37, 11.50).

**Table 2:** Independent associations of maternal overweight and GDM groups with offspring categorised as rapidly increasing skinfold thickness trajectory

Maternal exposure	*n*/*N* (%)	Model 1		Model 2	
		OR (95% CI)	*p*	OR (95% CI)	*p*
Maternal overweight					
Non-overweight	220/484 (45.45)	1		1	
Overweight	50/80 (62.50)	2.05 (1.25, 3.42)	0.005	2.04 (1.21, 3.48)	0.008
Maternal GDM					
Non-GDM	222/492 (45.12)	1		1	
GDM	48/72 (66.67)	2.65 (1.55, 4.65)	<0.001	2.95 (1.67, 5.34)	<0.001

Model 1: maternal age, education level (higher/lower), parity (primiparity/multiparity), maternal smoking (yes/no)

Model 2: Model 1 + paternal BMI (at OGTT), offspring breastfeeding status, paternal diabetes status (yes/no), offspring sex (male/female) + maternal overweight or maternal GDM

**Table 3:** Joint associations of both maternal overweight and GDM with offspring categorised as rapidly increasing skinfold thickness trajectory

Maternal groups	*n*/*N* (%)	Model 1		Model 2	
		OR (95% CI)	*p*	OR (95% CI)	*p*
Non-overweight and non-GDM	182/425 (42.82)	1		1	
Overweight and non-GDM	40/67 (59.70)	2.06 (1.12, 3.55)	0.008	1.99 (1.14, 3.53)	0.016
Non-overweight and GDM	38/59 (64.41)	2.67 (1.50, 4.87)	0.001	2.87 (1.56, 5.42)	0.001
Overweight and GDM	10/13 (76.92)	5.31 (1.54, 24.66)	0.015	6.90 (1.89, 33.32)	0.006

Model 1: adjusted for maternal age, education level (higher/lower), parity (primiparity/multiparity), maternal smoking (yes/no)

Model 2: adjusted for Model 1 + paternal BMI (at OGTT), offspring breastfeeding status, paternal diabetes status (yes/no), offspring sex (male/female)

LMM analysis (Table 4) in univariate, as well as fully adjusted, models revealed that children exposed to maternal overweight and GDM experienced the fastest increase in sum of skinfold thickness from birth to approximately 10 years old, compared with those born to normal-weight and non-GDM mothers (β 1.62; 95% CI 0.69, 2.54).

**Table 4:** Estimate regression parameters for LMM for interactions of maternal BMI and GDM exposure and offspring age with offspring sum of skinfold over time

Estimate regression parameters	Model 1		Model 2	
	β (95% CI)	*p*	β (95% CI)	*p*
Offspring age	2.59 (2.43, 2.75)	<0.001	2.57 (2.41, 2.73)	<0.001
Overweight and non-GDM	1.01 (−2.81, 4.82)	0.607	0.68 (−3.17, 4.52)	0.729
Non-overweight and GDM	1.13 (−3.12, 5.39)	0.634	0.96 (−3.03, 4.96)	0.640
Overweight and GDM	1.21 (−7.53, 9.96)	0.795	1.80 (−6.36, 9.97)	0.667
Maternal age	−0.43 (−0.65, −0.22)	<0.001	−0.45 (−0.66, −0.23)	<0.001
Higher education	−3.50 (−8.37, 1.36)	0.161	−4.97 (−10.22, 0.28)	0.067
Multiparity	2.49 (0.45, 4.54)	0.018	2.35 (0.31, 4.40)	0.026
Maternal smoking	1.94 (−7.60, 11.35)	0.671	−2.43 (−11.88, 7.01)	0.617
Paternal BMI			0.66 (0.38, 0.94)	<0.001
Paternal diabetes			0.37 (−4.72, 5.47)	0.888
Offspring sex, male			0.67 (−1.17, 2.51)	0.479
Offspring breastfeeding status			−1.85 (−3.69, −0.01)	0.052
Overweight and non-GDM×age	0.75 (0.32, 1.18)	0.001	0.77 (0.33, 1.21)	0.001
Non-overweight and GDM×age	0.58 (0.13, 1.03)	0.011	0.55 (0.11, 1.01)	0.015
Overweight and GDM×age	1.60 (0.67, 2.52)	0.001	1.62 (0.69, 2.54)	0.001

Model 1: Maternal age, education level (higher/lower), parity (primiparity/multiparity), maternal smoking (yes/no)

Model 2: Model 1 + offspring breastfeeding status, paternal BMI (at OGTT), paternal diabetes status, offspring sex

ESM Tables 5 and 6 outline the individual and combined correlations of maternal preconceptional BMI and GDM with childhood adiposity risk at ages 7 and 10. Our study further validates a dose–response correlation between maternal preconceptional BMI and adiposity risk, defined as skinfold thickness exceeding the 85th percentile, stratified by age and sex in our population, after adjusting for key covariates (ESM Figs 3, 4). A further analysis using BMI trajectory as the childhood adiposity measure revealed findings similar to the original results, with minor variations in the magnitude of effect estimates (ESM Tables 7, 8, ESM Fig. 5). Additionally, to address the potential impact of uneven measurement intervals, we performed a sensitivity analysis with more detailed age groups (ESM Fig. 6), which revealed similar trends and supported the robustness of our primary findings.

## Discussion

In an analysis of a prospective cohort from birth to early adolescence, we found that children born to mothers categorised as overweight and GDM had the highest odds of adverse adiposity outcomes, as measured by the sum of skinfold thickness. This indicates that the combination of maternal overweight and GDM places an exposed child at elevated risk of developing an increased growth pattern and higher adiposity.

Extensive evidence highlights the complex association between maternal metabolic condition and offspring health outcomes. The association between maternal GDM and adiposity in children has been extensively documented [11]. While adolescent offspring of women with GDM exhibit increased adiposity, the impact of early hyperglycaemic events in women who later develop GDM on fetal growth and development trajectories is rarely explored. Our findings support that maternal metabolic conditions during pregnancy are associated with offspring adiposity development, although the underlying mechanisms may involve multiple pathways including genetic predisposition, intrauterine environment and shared postnatal lifestyle factors [20]. Additionally, our analysis using continuous BMI showed that each unit increase in maternal preconceptional BMI was associated with 14% higher odds of offspring following a rapidly increasing skinfold thickness trajectory, suggesting a dose-dependent effect. According to Hanson and Gluckman, these effects can extend across generations, potentially creating cycles where maternal metabolic conditions influence offspring health, which in turn may affect subsequent generations [21]. Although maternal overweight and GDM combined showed a stronger association with offspring adiposity than either factor alone, formal testing did not support significant additive interaction, possibly due to the limited sample size in the joint exposure group. This suggests these conditions may influence offspring adiposity through complex biological mechanisms beyond simple additive effects. Interestingly, in another recent analysis from the HAPO Hong Kong field centre with an extended 18 year follow-up [22], we observed association between maternal glucose levels during pregnancy and offspring adiposity and glucose metabolism in young adulthood, highlighting persistence of this association, and suggesting that intrauterine exposure to maternal metabolic disorder may potentially programme offspring health trajectories, with effects persisting throughout development.

Our findings align with previous studies on the association between adverse maternal BMI factors and offspring adiposity trajectories. While several studies have explored the link between higher maternal BMI and increased growth in children, most have focused on weight trajectories during infancy, with fewer extending their analysis into childhood or early adolescence [23, 24]. A systematic review has indicated that trajectories characterised by excessive rapid gain, whether consistent or at specific time points, were associated with high maternal pre-pregnancy BMI [23]. Additionally, a meta-analysis suggests that the association of GDM with higher BMI in offspring is largely attributable to maternal BMI [5]. Similar findings were reported in the multinational HAPO Follow-up Study, which demonstrated that maternal obesity and GDM have independent and additive associations with childhood adiposity measures at ages 10–14 years [25]. While the findings from that study captured adiposity at specific ages, our longitudinal design at three time points allowed us to characterise the patterns of adiposity development from birth to early adolescence. Moreover, our study extends these findings to a mainly Asian population, where metabolic responses and adiposity patterns may differ from Western populations.

Interestingly, our analysis revealed different patterns between BMI and skinfold thickness trajectories in relation to maternal overweight and GDM. While skinfold thickness measurements showed significant joint effects, BMI trajectories demonstrated independent but not combined associations. This difference needs careful consideration given the different adiposity components these measures capture. BMI, while commonly used for screening adiposity in children due to its practicality and established clinical cut-offs, does not accurately represent body fat distribution [13]. A systematic review assessing the acceptability and ease of use of obesity measures in children found significant variation in the sensitivity of BMI for diagnosing both obesity and overweight [26]. In contrast, skinfold thickness specifically measures subcutaneous fat, providing a more direct assessment of adiposity changes. Thus, our study evaluated adiposity primarily through skinfold thickness, which offers more precise assessment of body fat compared with BMI. The joint effects in skinfold thickness but not BMI trajectories suggest maternal overweight and GDM might have combined effects on subcutaneous fat deposition while affecting overall body mass accumulation independently. Thus, children born to mothers with both conditions may have accelerated subcutaneous fat accumulation, not fully captured by BMI. This distinction matters as subcutaneous and visceral adiposity contribute differently to cardiometabolic risks. Our study highlights the need for comprehensive adiposity assessment in understanding the intergenerational effects. Future studies may benefit from multiple adiposity measures to better differentiate fat distribution patterns and their health implications.

Maternal BMI and offspring growth association involves multiple biological mechanisms. Developmental programming associated with obesity likely occurs throughout gestation and neonatal life. Persistent effects could be partially mediated through the reprogramming of organ and tissue structure and function via in utero epigenetic mechanisms [27]. Maternal obesity also influences offspring development through inflammatory mediators from adipose tissue and altered adipokine signalling [8]. Regarding maternal hyperglycaemia and offspring adiposity, several hypotheses have been proposed to explain the mechanisms linking maternal hyperglycaemia to adverse outcomes in offspring. One suggests that elevated maternal glucose levels lead to fetal hyperglycaemia, which in turn stimulates the fetal pancreas to produce insulin, promoting fetal growth and increasing the risk of obesity later in life [9]. Another hypothesis posits that hyperinsulinaemia during fetal development can alter brain development, leading to long-term changes in appetite and metabolism regulation [28]. Additionally, research indicates that exposure to GDM can significantly alter gene expression profiles in embryos, affecting critical developmental pathways [29]. While these pathways may interact, maternal obesity and GDM appear to impact offspring adiposity through distinct regulatory modifications. Overall effects include increased insulin resistance, fetal glucose exposure, disturbances in cardiometabolic traits and systemic low-grade inflammation [7, 30]. These findings highlight the multifactorial and multistage nature of obesity development, emphasising the need for a comprehensive approach to its prevention and management. Identifying the critical periods when these significant changes are initiated is essential for creating effective interventions [8].

Thus, interventions that may help to reduce childhood obesity are crucial. While epidemiological findings suggest the potential benefits of early pregnancy interventions, including maternal dietary guidance, moderate physical activity and glucose monitoring [31], several randomised controlled trials have shown limited effectiveness of such interventions on offspring outcomes [32, 33]. These interventions may have been limited by intervention timing, insufficient intensity or duration or inadequate targeting of specific mechanistic pathways. Our findings on adiposity trajectories suggest more precisely timed and targeted interventions might be necessary. While research suggests that breastfeeding may protect against obesity or diabetes in the offspring [34], our analysis showed persistent effects of maternal metabolic conditions after adjusting for breastfeeding status, emphasising the need for more effective strategies to break the intergenerational metabolic risk cycle.

The study’s novelty lies in its use of data from a longitudinal mother–child cohort from multiple assessment points across early life. We demonstrated that children born to mothers with overweight/obesity and GDM have a significantly higher likelihood of following a high and rising adiposity trajectory from birth to early adolescence, compared with children of mothers without these conditions. We also account for potential confounding factors, including paternal BMI and diabetes status at baseline, often overlooked in previous studies [10, 24]. The repeated measurements allowed for application and comparison of two widely used longitudinal data analysis methods to describe adiposity trajectories and their determinants. Our complementary analysis approach also provided distinct insights into adiposity development. LMMs provide individualised growth profiles against the mean, offering health professionals derived phenotypes such as the adiposity traits growth rates. GBTM, a person-centred data-driven process that assumes the population is composed of latent groups, captures more detailed information, especially the longitudinal relationships at the child level. Together, the analyses enhance our understanding of both population-wide trends and subgroup-specific patterns in the development of childhood adiposity.

This study has limitations. Attrition resulted in the loss of approximately 42.4% and 41.3% of mother–offspring dyads at 7 and 10 years old, respectively. To address potential attrition bias, we compared key characteristics between follow-up and lost to follow-up. While most characteristics showed minimal differences, mothers who continued participation were slightly older and had higher preconceptional BMI, which might affect the generalisability of our findings. We acknowledge that unmeasured factors could contribute to potential bias, including maternal lifestyle factors during pregnancy and early-life environmental exposures. While our trajectory analysis identified distinct patterns of adiposity development, the clinical implications need further validation. GBTM, while useful for identifying distinct developmental patterns, has limitations as results depend partly on investigator judgement during model selection. Our identified trajectories should be interpreted within the context of methodological decisions rather than as definitive, naturally occurring groups. Although statistical fit indices favoured more complex models, we selected a two-class model based on practical considerations regarding adequate subgroup sample size. Additionally, our study included sex only as a covariate rather than conducting sex-stratified analyses due to sample size limitations, which may ignore potential sex-specific effects. Future studies with longer follow-up would be valuable to examine how these trajectories relate to important clinical outcomes such as metabolic syndrome and diabetes in later life, with larger sample sizes that would allow for meaningful sex-stratified analyses. Another limitation is the reliance on recalled maternal weight and data obtained from chart reviews in previous reports. Notably, measurements of infant weight and length were not recorded between birth and 36 months, preventing a detailed analysis of early-life growth trajectories and their effects on adiposity at age 10 years. Also, our adiposity measurements were collected at uneven time intervals. The small sample sizes in both the maternal overweight and GDM groups may limit the generalisability and statistical power, particularly in adjusted analyses, and could potentially have led to model overfitting when multiple covariates were included. The study population comprised only Asian women from Hong Kong, further limiting the applicability of the results to other populations.

### Conclusion

Maternal overweight and GDM are both independently and jointly associated with adverse adiposity trajectories in offspring from birth to early adolescence. Our study findings suggest an important role of maternal overweight and GDM in predicting future adiposity in offspring. Understanding the independent and combined effects of these maternal factors on offspring obesity is essential for developing effective prevention strategies. Further research, particularly long-term interventional studies, is needed to determine whether modifications of maternal metabolic conditions could influence offspring adiposity development.

## Supplementary Information

Below is the link to the electronic supplementary material.

ESM (PDF 579 KB)

## Data Availability

Data collected for the study will not be made available to others.

## Abbreviations

AP: Attributable proportion due to interaction
BIA: Bioelectrical impedance analysis
DXA: Dual-energy x-ray absorptiometry
GBTM: Group-based trajectory modelling
GDM: Gestational diabetes mellitus
HAPO: Hyperglycemia and Adverse Pregnancy Outcome
LMM: Linear mixed-effect model
RCS: Restricted cubic spline
RERI: Relative excess risk due to interaction
S: Synergy index
