# Supplementary material for: Association of maternal overweight and gestational diabetes mellitus with offspring adiposity trajectory: from birth to early adolescence
Source: Diabetologia. 2025 Jun 17;68(10):2194–204. doi: 10.1007/s00125-025-06468-6 (PMC12423264; doi:10.1007/s00125-025-06468-6)
Supplement: Supplementary file 1 — ESM (PDF 579 KB) [file 125_2025_6468_MOESM1_ESM.pdf]

Contents

ESM Table 1. Evaluation indicator of Group-based trajectory model (GBTM) 2

ESM Table 2. Comparison of baseline characteristics between participants in the HAPO follow-up study at 7 years and those lost to follow-up 3

ESM Table 3. Comparison of baseline characteristics between participants in the HAPO follow-up study at 10 years and those lost to follow-up 4

ESM Table 4. Independent associations of maternal BMI or sum of glucose z score at OGTT with offspring categorized as rapidly increasing skinfold thickness trajectory 5

ESM Table 5. Independent associations of maternal BMI and glucose with continuous measures of sum of skinfold thickness (mm) 6

ESM Table 6. The OR (95%CI) of offspring adiposity for different maternal overweight and GDM category at each timepoint 7

ESM Table 7. Joint associations of both maternal overweight and GDM with offspring categorized as rapidly increasing BMI trajectory 8

ESM Table 8. Estimate regression parameters for LMM for interactions of maternal BMI and GDM exposure and offspring age with offspring BMI over time. 9

ESM Fig 1. Flow chart describing enrolment for mother-children dyads participating in HAPO study. 11

ESM Fig 2: Directed acyclic graph (DAG) of exposures, outcomes, and covariates in the study of offspring adiposity 12

ESM Fig 3. Dose–response relationship between maternal BMI and offspring adiposity at approximately 7 years according to GDM/non GDM. 13

ESM Fig 4: Dose–response relationship between maternal BMI and offspring adiposity at approximately 10 years according to GDM/non GDM. 14

ESM Fig 5. Offspring BMI trajectory categories from birth to early adolescence from the group-based trajectory model. 15

ESM Fig 6. Sensitivity analysis of offspring sum of skinfold thickness trajectory categories from birth to early adolescence using refined age groupings. 16

ESM Table 1. Evaluation indicator of Group-based trajectory model (GBTM)

Number of classes	APP				OCC				AIC	BIC	Participants per class (%)			
	G1	G2	G3	G4	G1	G2	G3	G4			1	2	3	4
1	1.00								13930.14	13951.88	564 (100.00)			
2	0.98	0.98			45.56	48.63			12357.94	12401.41	294 (52.13)	270 (47.87)		
3	0.96	0.95	0.94		35.36	61.73	30.92		11899.51	11975.58	246 (43.62)	133 (23.58)	185 (32.80)	
4	0.91	0.96	0.92	0.95	27.60	105.55	29.69	57.43	11640.37	11738.18	158 (28.01)	109 (19.33)	160 (28.37)	137 (24.29)

Abbreviation: AIC, Akaike information criterion, APP, Average posterior probability; BIC, Bayesian information Criterion; OCC, Odds of Correct Classification.

ESM Table 2. Comparison of baseline characteristics between participants in the HAPO follow-up study at 7 years and those lost to follow-up

	HAPO follow-up at 7 years (n=961)	HAPO lost to follow-up at 7 years (n=636)	*P*
**Maternal characteristics**			
Age, year	31.3±4.6	30.3±5.1	<0.001
Preconceptional BMI (kg/m^2)	20.9±2.9	20.3±2.8	<0.001
Higher education level	901 (96.3)	609 (97.6)	0.187
Parity, primiparity	574 (59.7)	403 (63.4)	0.160
Family history of diabetes	335 (34.9)	202 (31.8)	0.219
Smoker	16 (1.7)	18 (2.8)	0.161

ESM Table 3. Comparison of baseline characteristics between participants in the HAPO follow-up study at 10 years and those lost to follow-up

	HAPO follow-up at 10 years (n=564)	HAPO lost to follow-up at 10 years (n=1033)	*P*
**Maternal characteristics**			
Age, year	31.5±4.6	30.5±5.0	<0.001
Pre-conceptional BMI (kg/m^2)	21.1±2.9	20.4±2.8	<0.001
Higher education level	527 (96.2)	983 (97.1)	0.377
Parity, primiparity	327 (58.0)	650 (62.9)	0.060
Family history of diabetes	206 (36.5)	331 (32.0)	0.079
Smoker	6 (1.1)	28 (2.7)	0.046
**Paternal Characteristics During HAPO Study**			
Paternal BMI at original OGTT (kg/m^2)	23.3±3.3	23.2±3.3	0.840
Paternal diabetes	18 (3.2)	7 (1.8)	0.204

ESM Table 4. Independent associations of maternal BMI or sum of glucose z score at OGTT with offspring categorized as rapidly increasing skinfold thickness trajectory

Maternal exposure	Model 1		Model 2	
	OR (95%CI)	*p*	OR (95%CI)	*p*
Maternal preconceptional BMI	1.17 (1.10, 1.25)	<0.001	1.14 (1.07, 1.23)	<0.001
Maternal sum of glucose z score at original OGTT	1.15 (1.06, 1.25)	<0.001	1.14 (1.04, 1.24)	0.004

The sum of glucose z scores was calculated by glucose level at OGTT, subtracting the mean glucose level, dividing by the SD for each time point, and summing these individual “z scores”.

Model 1: Adjusted for maternal age, education level (higher/lower), parity (primiparity/multiparity), and maternal smoking (yes/no).

Model 2: Adjusted for Model 1 + paternal BMI (at original OGTT), paternal diabetes status (yes/no), offspring breastfeeding status (yes/no), offspring sex (male/female), and maternal sum of glucose z score at OGTT or BMI.

ESM Table 5. Independent associations of maternal BMI and glucose with continuous measures of sum of skinfold thickness (mm)

Offspring Outcome	Maternal BMI		Maternal glucose sum of z-scores	
	β (95%CI)	*p*	β (95%CI)	*p*
**At 7 years**				
Model 1	1.25 (0.93, 1.57)	<0.001	0.91 (0.45, 1.37)	<0.001
Model 2	1.23 (0.90, 1.56)	<0.001	0.72 (0.26, 1.17)	0.002
Model 3	1.21 (0.88, 1.55)	<0.001	0.65 (0.18, 1.12)	0.007
**At 10 years**				
Model 1	1.82 (1.20, 2.43)	<0.001	1.75 (0.95, 2.54)	<0.001
Model 2	1.70 (1.08, 2.32)	<0.001	1.32 (0.52, 2.12)	0.001
Model 3	1.58 (0.97, 2.19)	<0.001	1.31 (0.50, 2.12)	0.002

β represents differences in each outcome for maternal BMI higher by 1.0 kg/m^2 and maternal glucose sum of z-scores higher by 1 unit.

Model 1: Maternal Age.

Model 2: Model 1 + maternal GDM/maternal BMI+ education level (higher/lower), parity (primiparity/multiparity), and maternal smoking (yes/no).

Model 3: Model 2 + paternal BMI at OGTT, paternal diabetes (yes/no), offspring breastfeeding status (yes/no), offspring age, and offspring sex (male/female).

For outcomes at 7 years, model 3 additionally adjusted for offspring’s exercise frequency (never /regular /frequent).

ESM Table 6. The OR (95%CI) of offspring adiposity for different maternal overweight and GDM category at each timepoint

**Offspring adiposity traits**	Non-overweight and non-GDM	Overweight and non-GDM	Non-overweight and GDM	Overweight and GDM
Sum of skinfold thickness> 85th percentiles at mean age of 7 years old	1	3.67 (2.11, 6.28)	1.17 (0.62, 2.11)	3.68 (1.36, 9.25)
Sum of skinfold thickness > 85th percentiles at mean age of 10 years old	1	3.13 (1.57, 6.11)	2.01 (0.89, 4.26)	6.88 (1.82, 25.19)
Sum of skinfold thickness increase between 7- to 10- years old > 50th percentiles	1	1.64 (0.93, 2.91)	2.48 (1.35, 4.65)	8.35 (2.06, 56.53)

Model were adjusted for maternal age, education level (higher/lower), parity (primiparity/multiparity), maternal smoking (yes/no), paternal BMI (at original OGTT), paternal diabetes (yes/no), and offspring breastfeeding status (yes/no).

Adiposity traits at 7 years were additionally adjusted for offspring exercise frequency (never /regular /frequent).

ESM Table 7. Joint associations of both maternal overweight and GDM with offspring categorized as rapidly increasing BMI trajectory

Maternal groups	n/N (%)	Model 1		Model 2	
		OR (95%CI)	*p*	OR (95%CI)	*p*
Non-overweight and non-GDM	126/425 (29.65)	1		1	
Overweight and non-GDM	30/67 (44.78)	1.97 (1.15, 3.37)	0.013	2.04 (1.16, 3.60)	0.013
Non-overweight and GDM	26/59 (44.07)	2.06 (1.15, 3.67)	0.014	2.20 (1.18, 4.08)	0.012
Overweight and GDM	6/13 (46.15)	2.07 (0.63, 6.62)	0.216	2.86 (0.82, 9.71)	0.090

Model 1: maternal age, education level (higher/lower), parity (primiparity/multiparity), and maternal smoking (yes/no).

Model 2: Model 1 + paternal BMI (at original OGTT), paternal diabetes (yes/no), offspring breastfeeding status (yes/no), and offspring sex (male/female).

ESM Table 8. Estimate regression parameters for LMM for interactions of maternal BMI and GDM exposure and offspring age with offspring BMI over time.

	Model 1		Model 2	
	β (95%CI)	*p*	*β* (95%CI)	*p*
Offspring's age	0.39 (0.36, 0.41)	<0.001	0.38 (0.36, 0.41)	<0.001
Overweight and non-GDM	0.39 (-0.20, 0.99)	0.197	0.36 (-0.23, 0.95)	0.236
Non-overweight and GDM	0.73 (0.11, 1.35)	0.023	0.72 (0.11, 1.33)	0.022
Overweight and GDM	0.56 (-0.72, 1.85)	0.392	0.78 (-0.47, 2.04)	0.223
Maternal age	-0.09 (-0.12, -0.05)	<0.001	-0.09 (-0.12, -0.06)	<0.001
Higher education	-0.47 (-1.25, 0.30)	0.236	-0.78 (-1.60, 0.04)	0.065
Multiparity	0.40 (0.08, 0.72)	0.017	0.35 (0.03, 0.67)	0.034
Maternal smoking	0.21 (-1.20, 1.62)	0.771	-0.42 (-1.90, 1.05)	0.580
Offspring breastfeeding status	NA	NA	-0.19 (-0.48, 0.10)	0.197
Paternal BMI	NA	NA	0.12 (0.08, 0.15)	<0.001
Paternal diabetes	NA	NA	0.03 (-0.76, 0.83)	0.932
Offspring sex, male	NA	NA	0.60 (0.32, 0.89)	<0.001
Overweight and non-GDM × age	0.09 (0.02, 0.15)	0.010	0.09 (0.02, 0.15)	0.009
Non-overweight and GDM × age	0.01 (-0.06, 0.08)	0.822	0.01 (-0.06, 0.07)	0.871
Overweight and GDM × age	0.13 (-0.01, 0.27)	0.063	0.14 (0.00, 0.28)	0.049

Model 1: Adjusted for maternal age, education level (higher/lower), parity (primiparity/multiparity), and maternal smoking (yes/no).

Model 2: Model 1 + offspring breastfeeding status (yes/no), paternal BMI (at original OGTT), paternal diabetes (yes/no), offspring sex (male/female).

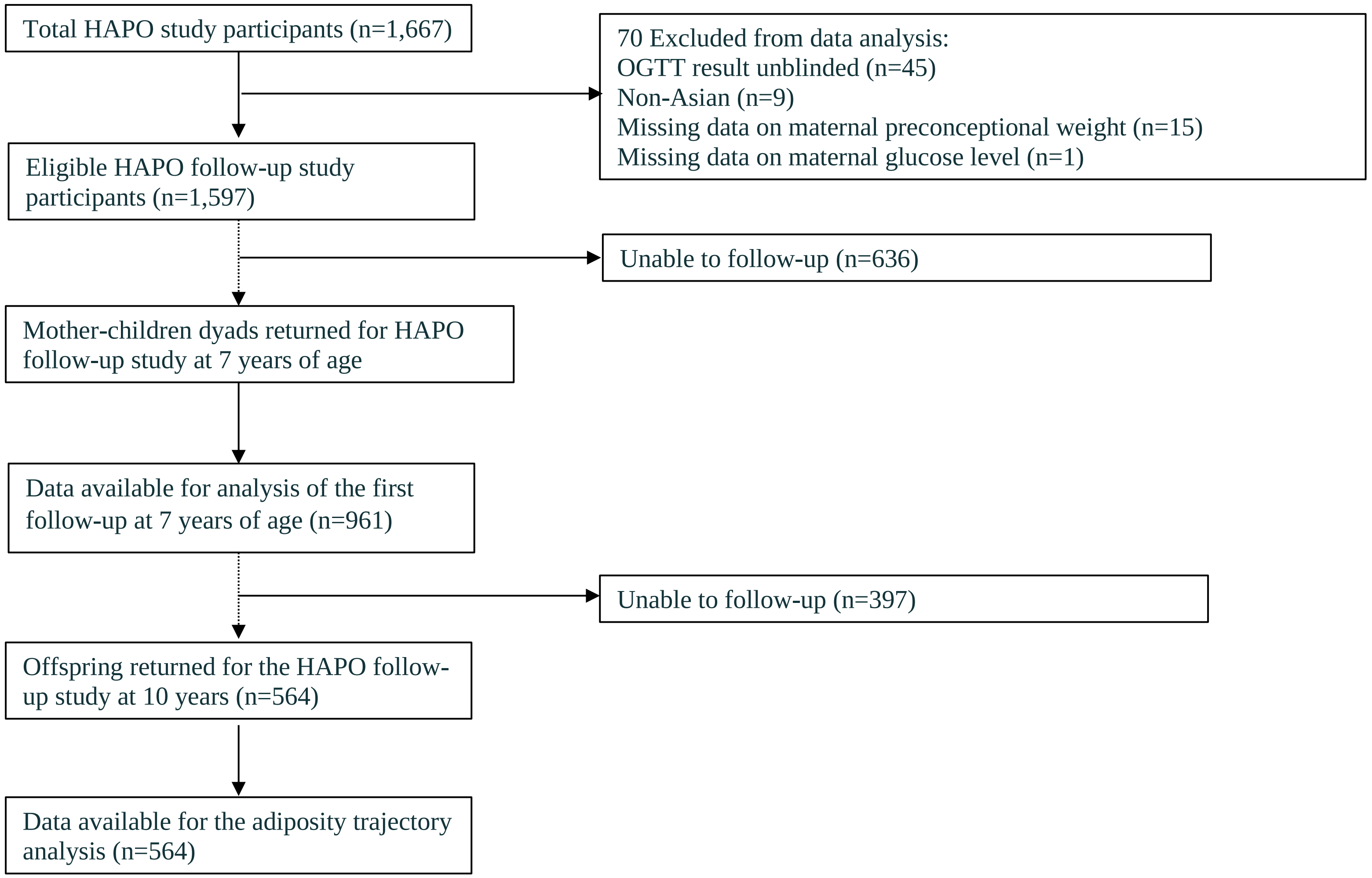

ESM Fig 1. Flow chart describing enrolment for mother-children dyads participating in HAPO study.

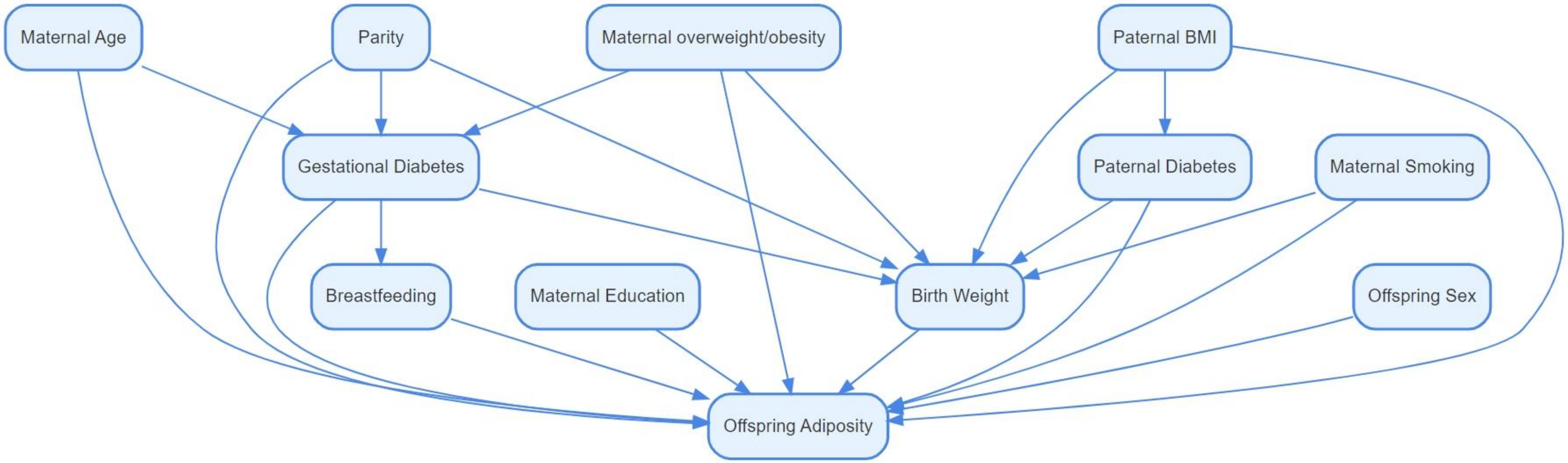

ESM Fig 2: Directed acyclic graph (DAG) of exposures, outcomes, and covariates in the study of offspring adiposity

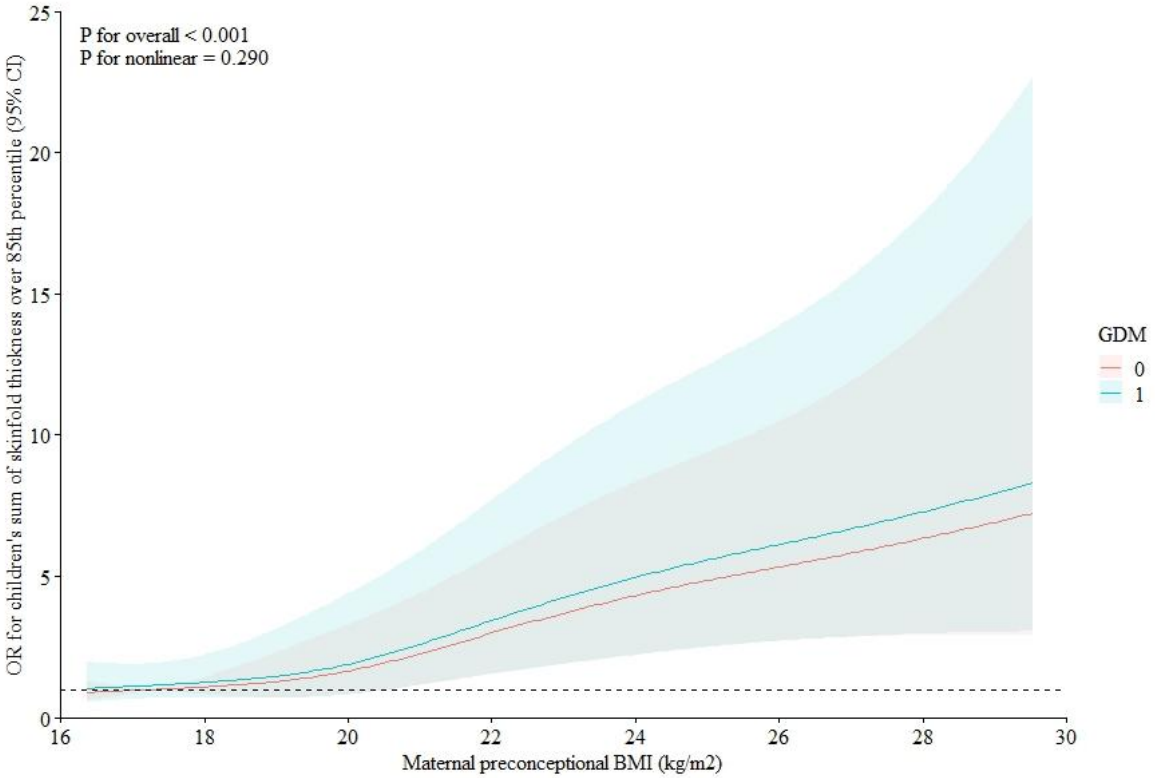

ESM Fig 3. Dose–response relationship between maternal BMI and offspring adiposity at approximately 7 years according to GDM/non GDM.

The red curves represent adjusted ORs for maternal BMI without further developing GDM based on restricted cubic splines (RCS) with knots at the 5th, 35th, 65th, and 95th percentiles of maternal BMI. The blue curves represent adjusted ORs for maternal BMI among women further developing GDM. Shadow shows the 95% CIs for RCS of maternal BMI.

Model were adjusted for maternal age, education level (higher/lower), parity (primiparity/multiparity), maternal smoking (yes/no), paternal BMI (at original OGTT), offspring breastfeeding status (yes/no), paternal diabetes (yes/no), and offspring exercise frequency (never /regular /frequent).

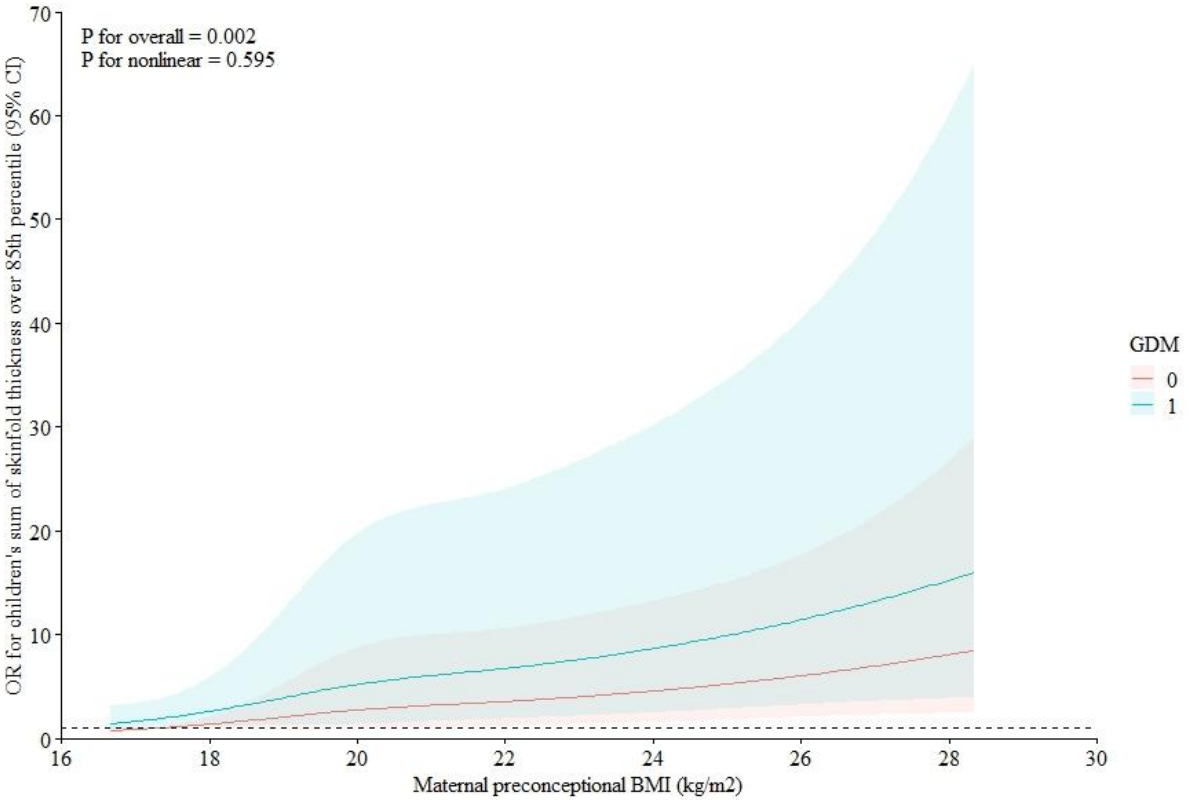

ESM Fig 4: Dose–response relationship between maternal BMI and offspring adiposity at approximately 10 years according to GDM/non GDM.

The red curves represent adjusted ORs for maternal BMI without further developing GDM based on restricted cubic splines (RCS) with knots at the 5th, 35th, 65th, and 95th percentiles of maternal BMI. The blue curves represent adjusted ORs for maternal BMI among women further developing GDM. Shadow shows the 95% CIs for RCS of maternal BMI.

Model were adjusted for maternal age, education level (higher/lower), parity (primiparity/multiparity), maternal smoking (yes/no), paternal BMI (at original OGTT), offspring breastfeeding status (yes/no), and paternal diabetes status (yes/no).

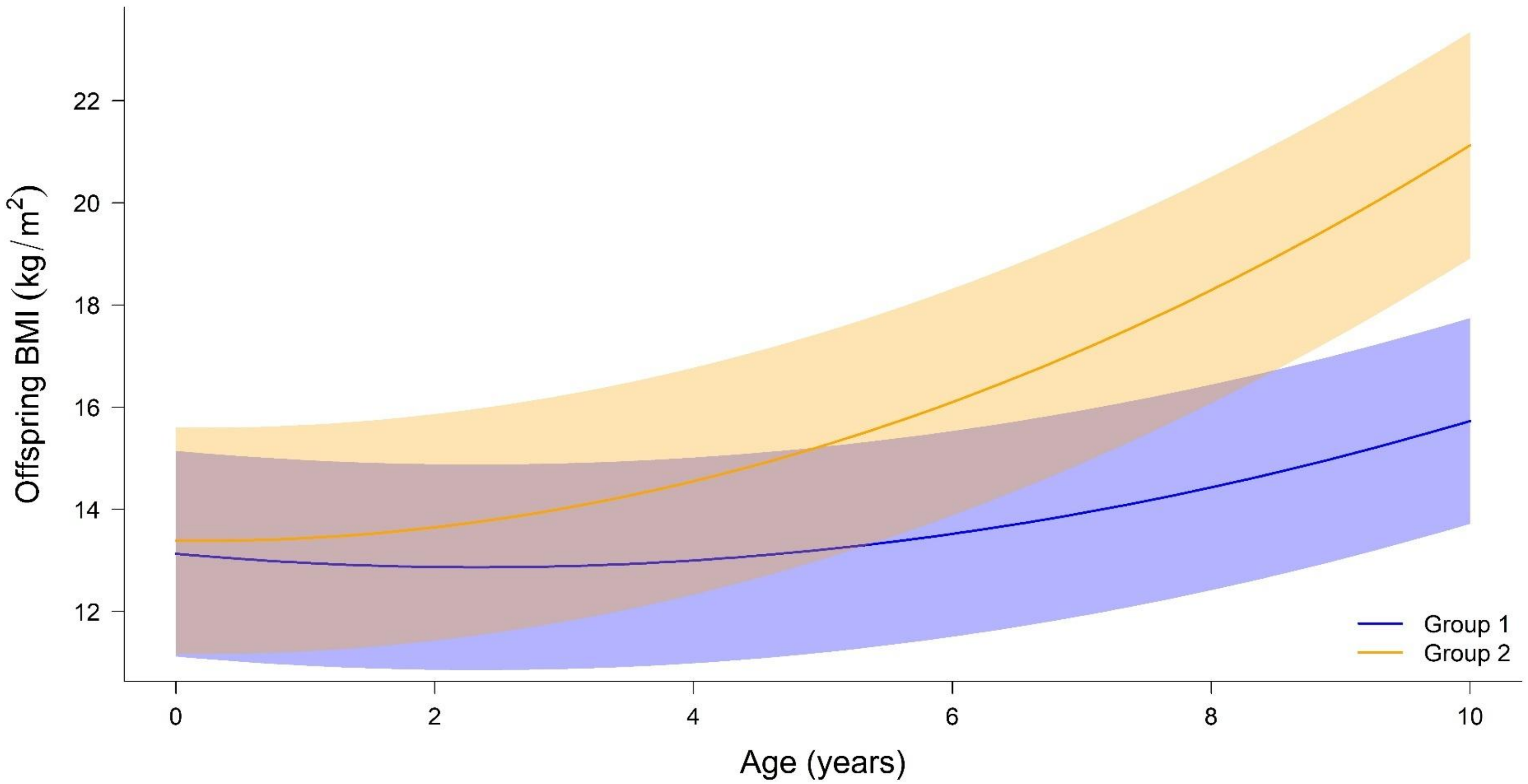

ESM Fig 5. Offspring BMI trajectory categories from birth to early adolescence from the group-based trajectory model.

Legend: Two latent trajectory groups with quadratic functions were identified. The blue curve (group 1, n=376) is categorised as the slowly increasing BMI trajectory, and the orange curve (group 2, n=188) as categorised as the rapidly increasing BMI trajectory. Shadow indicates the corresponding 95% CIs.

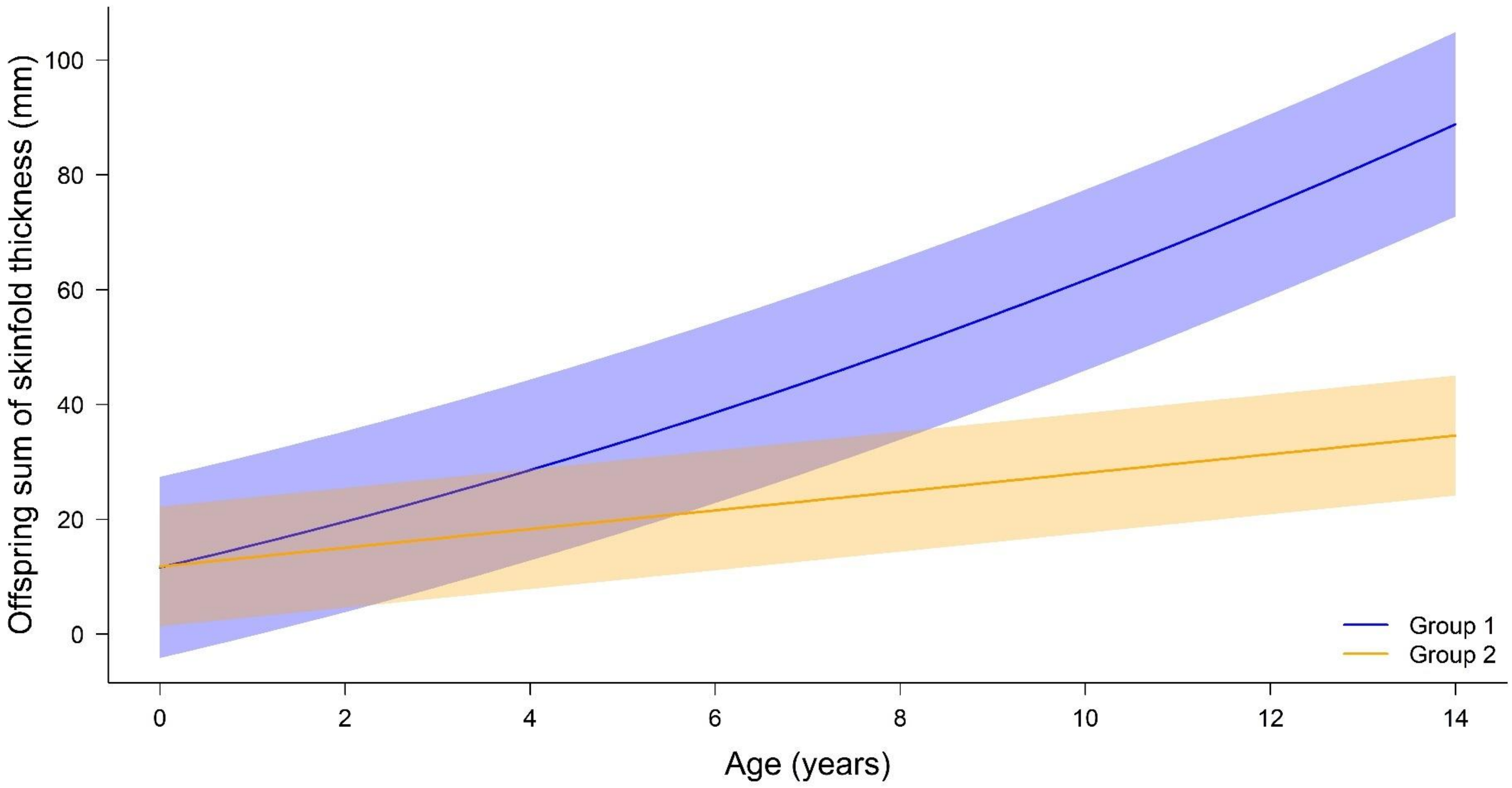

ESM Fig 6. Sensitivity analysis of offspring sum of skinfold thickness trajectory categories from birth to early adolescence using refined age groupings.

Legend: Two latent trajectory groups with quadratic functions were identified. The model was built using the offspring's more detailed age in completed years. The blue curve (group 1, n=180) represents a rapid increase in skinfold thickness, while the orange curve (group 2, n=384) shows a slow increase. Shadow indicates the corresponding 95% CIs.
